# Differential change in alcohol consumption during the COVID-19 pandemic: the role of loneliness, socialization, and mental well-being

**DOI:** 10.3389/fpsyt.2024.1236410

**Published:** 2024-03-01

**Authors:** Mohamed S. Mohamed, Gull Rukh, Sofia Vadlin, Susanne Olofsdotter, Cecilia Åslund, Helgi B. Schiöth, Kent W. Nilsson

**Affiliations:** ^1^ Center for Clinical Research, Uppsala University, Västmanland County Hospital Västerås, Västerås, Sweden; ^2^ Functional Pharmacology and Neuroscience, Department of Surgical Sciences, Uppsala University, Uppsala, Sweden; ^3^ Department of Psychology, Uppsala University, Uppsala, Sweden; ^4^ Department of Public Health and Caring Sciences, Uppsala University, Uppsala, Sweden; ^5^ School of Health, Care and Social Welfare, Division of Public Health Sciences, Mälardalen University, Västerås, Sweden

**Keywords:** alcohol consumption, COVID-19, social isolation, loneliness, mental health

## Abstract

**Introduction:**

The COVID-19 pandemic led to a surge in mental health issues and psychological distress, disruption to work/studying conditions, and social isolation particularly among young adults. Changes in these factors are differentially associated with alcohol use. Moreover, the relationship between these factors are bidirectional and may have fluctuated throughout the different phases of the pandemic. However, studies focusing on young adults had conflicting results, short follow-up periods, and lacked comprehensive data to describe underlying mechanisms.

**Methods:**

1067 young adults participated in repetitive measures termed wave 4 (2021) of the Survey of Adolescent Life in Västmanland Cohort “SALVe” Cohort. Of these, 889 also completed pre-pandemic measurements termed wave 3 (2018). Participants completed the Alcohol Use Disorders Identification Test (AUDIT) to evaluate alcohol consumption and harmful use. Cross-sectional associations between perceived changes in alcohol use and shift in individual, mental health, and work environment factors were examined using Chi-square tests. Logistic regression was utilized to identify pre-pandemic predictors of harmful consumption during the pandemic.

**Results:**

Harmful consumption decreased only in females following the COVID-19 pandemic. Participants who reported increased feelings of depression, anxiety, and loneliness were more likely to increase their alcohol use. Interestingly, the subgroup who felt less lonely and met their friends more often, as well as those who continued working/studying from their regular workplace also had an increased likelihood of higher consumption. Only pre-pandemic ADHD and delinquency symptoms predicted harmful alcohol consumption following the pandemic.

**Conclusion:**

Females reduced harmful alcohol consumption during the COVID-19 pandemic. While those who suffered the burden of social isolation and distress were more likely to increase their alcohol use, young adults who felt less lonely and met their friends more often also had a similar outcome. The relationship between loneliness and alcohol consumption among young adults is influenced by the social factors that may be facilitated by drinking.

## 1 Introduction

The COVID-19 pandemic prompted an unprecedented public health response worldwide in hopes of mitigating the spread of the novel coronavirus. While the policies were effective in slowing the spread of the virus, the response has also led to an increase in personal distress and social isolation (1). Moreover, a surge in mental health issues was observed during the period (2–4). Those with pre-existing psychiatric disorders had worsening symptoms (5). Specifically, young adults were particularly negatively impacted causing a deterioration in mental health and well-being (6). Such factors may be differentially associated with greater alcohol use and related harm especially among vulnerable groups. Psychological stress among young adults may lead to greater alcohol use and misuse (7). Likewise, exposure to disastrous/catastrophic events (hurricanes, outbreaks, terrorist attacks) that threaten physical/emotional well-being is associated with increased alcohol consumption (8). Alcohol use may also constitute a maladaptive coping strategy in times of distress and social isolation (9, 10). Furthermore, consumption may increase due to a reduction in drinking consequences. The shift to remote studying/work due to the pandemic may lead to a reduction of constraints related to alcohol use such as visible hangover symptoms and mild intoxication causing an increase in consumption (11). On the other hand, alcohol consumption may decrease during the pandemic due to reduced availability. Specifically, the closure of bars and on-premise consumption sites due to lockdowns and social distancing policies may cause an immediate reduction in alcohol use (10). The shift in the social context of drinking may also influence consumption as young adults drink more in social contexts and at parties than alone or at home (12, 13). Hence, an alternative scenario of reduced alcohol consumption may occur during the pandemic, especially in groups with social motives for drinking. These two plausible scenarios may differ or co-occur among young adults due to differences in both the impact on individuals and the policies taken in different phases during the pandemic.

During the first months of the pandemic, a study in Australia, a country that had stricter policies, reported a decrease in alcohol use especially among younger adults (14, p. 19). Similarly, a reduction in alcohol-related harm was reported during the first year of the pandemic independent of sex (15). In contrast, a study in the U.S. for the first six months found younger age to be associated with greater consumption (16). Moreover, those who experienced strict policies were more likely to fall into harmful use compared to those under no restrictions (16). The two early studies, however, differed in methodology and demographics which might explain the discrepancies. Systematic reviews and meta-analysis of all age groups showed that roughly equal percentages indicated an increase in consumption compared to those demonstrating a decrease in use (17). However, young adults were more likely to increase their use with both individual and mental health factors implicated in consumption change (17). Notably, the review showed that most studies relied on retrospective data and concluded the need for longitudinal data (17). In contrast, a meta-analysis focusing on Europe communicated that a decline in use and frequency was more common except for heavy drinkers who maintained their use (18). Young adults have also been reported to decrease their binge drinking with unemployment and loneliness moderating the consumption change (19). On the other hand, no association between changes in drinking and changes in depression or anxiety was reported during the first year (15). The study however is limited by the small sample size and the first phases of the pandemic for measurement in contrast to the systematic review. In addition, adolescents with ADHD were reported to have increased consumption compared to those without ADHD (20). On the other hand, contradicting results on the role of impulsivity were reported (21, 22).

Policies to contain the COVID-19 pandemic were relaxed and restricted several periods and alcohol use may have fluctuated accordingly (23). The relationship between alcohol use and mental health is also bidirectional and higher use might lead to a worsening of symptoms (24, 25). Many previous studies had short following periods that could not capture the overall change throughout the pandemic (17, 18). To address the gap, the present study pursued three important questions by utilizing the comprehensive longitudinal data available in the “Survey of Adolescent Life in Västmanland Cohort” (SALVe cohort) project 1) what is the overall change in alcohol use among young adults throughout the different phases of the pandemic, 2) What are the pre-pandemic mental health issues that could predict hazardous use during the pandemic, and 3) Which shifts (in demographics, work/study, social life, and mental health) during the pandemic are associated with changes in alcohol use during the pandemic to understand the relationship between a shift in these factors and alcohol use.

## 2 Materials and methods

### 2.1 Study design

**Figure 1:**
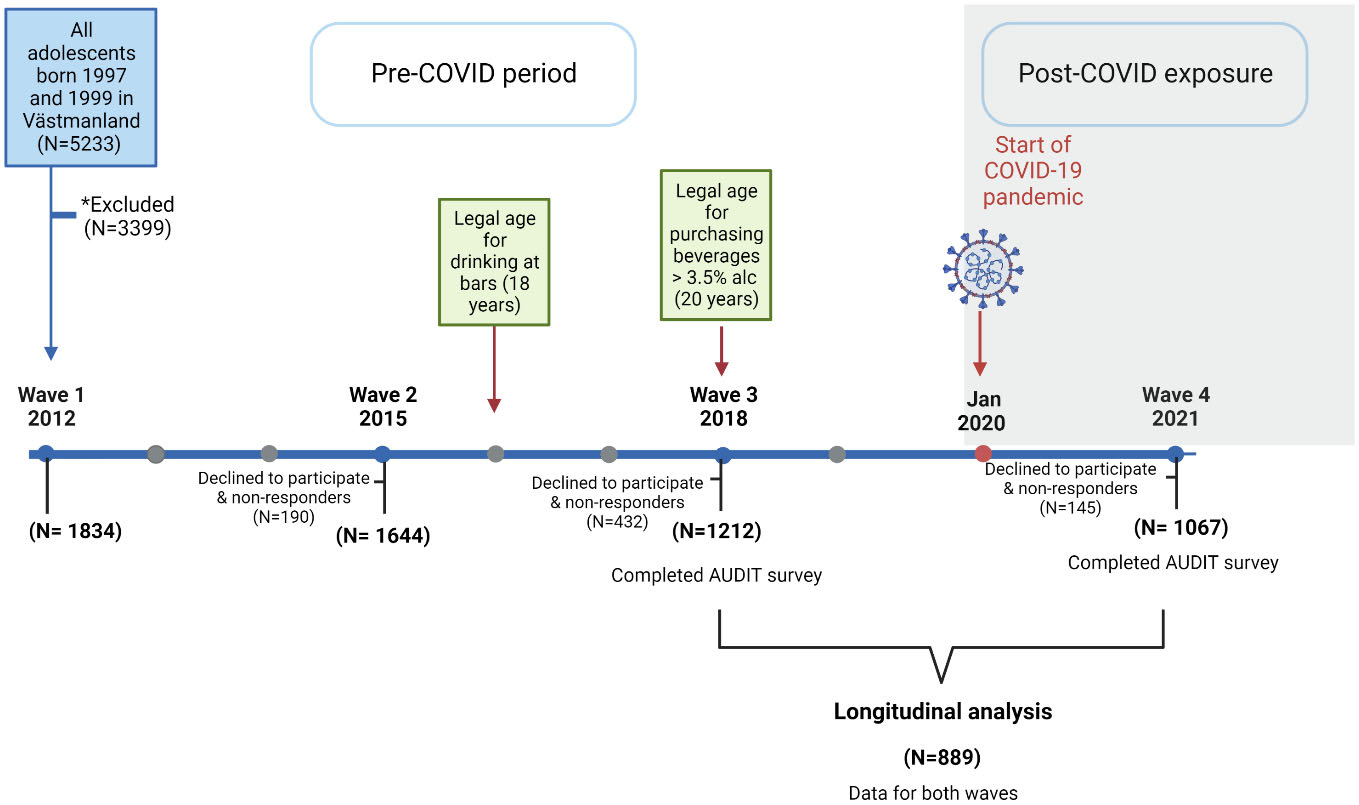
Study design flowchart: All adolescents born in 1997 and 1999 were invited to participate in wave 1 (2012). Crosstabulations and descriptive analysis were performed for wave 4 responders (N=1067) while longitudinal analysis was performed for those who completed surveys for the measurements used in this study in both waves (N=889). *Excluded due to language difficulties, severe mental disabilities or illness, not living in Västmanland in 2012, non-responders, declined to participate. AUDIT, Alcohol Use Disorders Identification Test.

The SALVe cohort project started in 2012 with data at four time points collected in the cohort represented by wave 1 to wave 4 (**Figure 1**). Adolescents born in 1997 or 1999, and their parents, who are living in Västmanland county in Sweden were contacted by regular mail and asked to participating in the study. The initial study population was 1863 adolescents (55.4% girls). In this study, data collected at two time points before and following the COVID-19 pandemic (Wave 3: November 2018, wave 4: Oct 2021) was used to describe the trend of alcohol consumption and hazardous use throughout the period. In total, 1067 participants (mean age 23, 64.3% females) who completed both waves were included. Wave 3 was considered as the pre-COVID-19 period and wave 4 as the post-COVID-19 period.

### 2.2 Measurements

Participants completed the Alcohol Use Disorders Identification Test (AUDIT) at the two time points (**Figure 1**). The AUDIT survey is a widely-used screening tool for alcohol use disorders and consists of 10 questions used to assess alcohol consumption and alcohol-related harm (26, 27). The sum of the first 3 AUDIT questions was used to evaluate alcohol consumption using the AUDIT-C scale (28). Both AUIDT and AUDIT-C are good validated measurements for adolescents and young adults (29, 30). AUDIT-C questions had a scale ranging from 0 to 4 and a total sum range of 0-12. Drinking frequency was categorized as Never, once a month or more seldom, 2-4 times a month, 2-3 times a week, and 4 times/week or more. Drinks per occasion were categorized as 1-2, 3-4, 5-6, 7-9, and 10 drinks or more. Binge drinking (heavy episodic drinking; HED), defined as 6 drinks or more, was categorized as Never, less often than once a month, every month, every week, and daily or almost every day. The sum of questions 4-10 was used to create an index for alcohol-related harm and the index was used to assess cross-sectional associations (e.g “How often in the past year have you stopped doing something you should because you were drinking?”, “How often in the past year have you had feelings of guilt or remorse because of your drinking?”, “How often in the past year have you been drinking so that the next day you did not remember what you said or did?”).

AUDIT score was calculated by adding the sum of the AUDIT-C and the alcohol-related harm indexes. Higher AUDIT scores indicate higher hazardous/harmful consumption. We used the WHO recommendations for the categorization of hazardous alcohol use (male ≥ 8 and female ≥ 6) to identify pre-pandemic predictors. Finally, we added a question on perceived changes in alcohol consumption during the COVID-19 pandemic “Have your alcohol drinking habits changed since the start of the corona pandemic?? with a response scale from 0 to 3 “0= Yes, increased, 1= No, decreased, 2= Unchanged, 3= I do not drink alcohol, neither now nor before the start of the corona pandemic”. Internal consistency for AUDIT in this study was measured by Cronbach’s alpha and was α= 0.812 for wave 4 and α= 0.811 for wave 3.

### 2.3 Independent variables

#### 2.3.1 Demographics

Age was coded by year of birth (1997 & 1999) and sex was coded by 1= male, 2= female. Questions regarding perceived changes due to the COVID-19 pandemic in employment, working hours, income, and shifts to remote working were added at wave 4 “Has your situation changed since the start of the COVID-19 pandemic”.

#### 2.3.2 Social factors

Participants were asked about perceived changes in their relationship status, meetings with friends/family, and feelings of loneliness since the start of the COVID-19 pandemic to evaluate the trend of alcohol consumption “Has your situation changed since the start of the COVID-19 pandemic”. Changes were categorized as More often, Unchanged, and Less often and coded as 0, 1, and 2 respectively.

#### 2.3.3 Mental health

Participants completed surveys on self-rated symptoms of depression, anxiety, Attention deficit hyperactivity disorder (ADHD), drug use, delinquency symptoms (wave 3), and psychotic-like experiences (wave 3).

Depression symptoms was measured by the Depression Self-Rating Scale (DSRS). The scale, developed and validated originally in Sweden, covers the DSM-IV criteria for depression in adolescents and is a widely used diagnostic tool for measurement of depressive symptoms in epidemiological studies with good sensitivity and specificity (31, 32). Internal consistency was measured by Cronbach’s alpha and DSRS showed high consistency in both waves (α= 0.857 in wave 4 and α= 0.865 in wave 3).

Anxiety symptoms were assessed using the self-rating Adult Anxiety Scale (AAS-15). The AAS-15 can be described as an age-adjusted version of the well-studied Spence Children’s Anxiety Scale (SCAS) and is comprised of 15 items to assess symptoms of anxiety categories (panic, generalized anxiety, and social phobia) in adults (Spence, 2017. The Adult Anxiety Scale-15 (AAS-15), self-report version. Personal communication)^1^. In the SALVe cohort, the AAS-15 has shown good psychometric properties in a pilot study. The AAS-15 internal consistency was excellent and ranged from (0.86-0.93), and the scale had good discriminative validity against depressive symptoms (DSRS). Confirmatory Factor Analysis (CFA) indicated good construct validity for AAS-15 subscales (Unpublished data). Cronbach’s alpha was used to measure AAS-15 internal consistency and was (α= 0.933 in wave 4 and α= 0.928 in wave 3).

ADHD symptoms were evaluated using the ADHD self-report scale (ASRS). The ASRS is a self-rating scale to assess symptoms of ADHD with good reliability (33). The ASRS had high internal consistency in this study when measured by Cronbach’s alpha (α= 0.927 in wave 4 and α= 0.902 in wave 3).

Drug use was assessed using the Drug Use Disorders Identification Test (DUDIT). The DUDIT is an 11-item self-rating scale to evaluate substance abuse with high sensitivity and reliability (34). Internal consistency for the DUDIT in this study was (α= 0.881 in wave 4 and α= 0.889 in wave 3) using Cronbach’s alpha measurement.

Delinquency symptoms were measured using a questionnaire as previously described (35). Participants were asked to rate the frequency of conduct problems on a five-point scale (0 = never, 1 = once, 2 = 2–4 times, 3 = 5–10 times, and 4 = >10 times). Higher scores indicated more intense delinquency symptoms/conduct problems. Cronbach’s alpha was α= 0.702 in wave 3. The questionnaire was not used at wave 4 as participants developed into young adults.

Psychotic-like symptoms were assessed with a self-report scale used for early detection in children and adolescents as previously described (36). The scale consists of nine items with a scale range as follows: 0 =Never, 1 = sometimes, and 2 = often. The scale showed high internal consistency using Cronbach’s alpha α= 0.797 in wave 3. Similar to delinquency symptoms, the scale was not used at wave 4 due to the participant’s development.

Finally, a separate question was added along the DSRS and AAS-15 surveys on perceived changes in feelings of depression/anxiety following the COVID-19 pandemic [Has your feelings of depression/anxiety changed since the start of the COVID-19 pandemic? (last 12 months)] at wave 4. The question had a response scale from 0 to 3 (0= Yes increased, 1= Yes, decreased, 2= Unchanged, 3= Never had depression/anxiety before or after the pandemic).

### 2.4 Statistical analysis

We employed a multifaceted statistical approach to comprehensively address the research questions posed in this study. For descriptive statistics, the mean, standard deviation, and percentages were calculated. Sex-stratified analysis for alcohol consumption and related harm between the pre-pandemic (wave 3) and the post-pandemic (wave 4) periods was done and changes between the two time-points were tested using the paired sample t-test.

For cross-sectional associations, crosstabulations were performed using chi-square tests. This allowed us to explore the relationships between perceived changes in demographics, social factors, and mental health with changes in alcohol consumption during the COVID-19 pandemic. In addition, a univariate general linear model analyses were performed to examine cross-sectional associations with alcohol-related harm at wave 4 controlled for sex.

Finally, logistic regression using the WHO classification for harmful use was utilized to identify pre-pandemic psychiatric traits that predicted hazardous use following the pandemic adjusting for sex (Q3). Additionally, we run the analyses by adding all the predictors in the same model to test the association of each predictor independent of the rest of the traits. Statistical analyses were performed using the Statistical Package for Social Sciences (SPSS version 26) and a p-value < 0.05 was considered significant.

### 2.5 Ethical considerations

The study was approved by the Ethical Review Board in Uppsala, Dnr: 2012/187, and in accordance with the Declaration of Helsinki. All participants provided written informed consent to participate in the study.

## 3 Results

### 3.1 Descriptive attributes of wave 4 and wave 3 of the SALVe cohort

The mean age of participants is 23 years in the fourth wave. Females constituted 64.2% of the sample at wave 4. The mean alcohol consumption and alcohol-related harm scores decreased in the post-COVID-19 group in both sexes. The mean score in consumption decreased from 4.1 to 3.89 in males and from 3.63 to 3.47 in females. Alcohol-related harm reduction was relatively more apparent in females where the mean decreased by 0.27 to 1.44 in wave 4 compared to a 0.05 decrease in males since the start of the COVID-19 pandemic. Similarly, the mean of the AUDIT total score decreased by 0.43 in females in contrast to a 0.2 reduction in males. However, the reduction in total AUDIT scores in females was the only significant change following the COVID-19 pandemic, p = 0.01 (**Table 1**).

**Table 1:** Descriptive statistics of alcohol use in the SALVe cohort before and after the COVID-19 pandemic stratified by sex.

Characteristics	Males n= 380			Females n= 687		
	Wave 3 (Pre-pandemic) M (SD)	Wave 4 (Post-pandemic) M (SD)	*P*	Wave 3 (Pre-pandemic) M (SD)	Wave 4 (Post pandemic) M (SD)	*P*
Alcohol consumption	4.1 (2.95)	3.8 9 (2.64)	1.0	3.63 (2.47)	3.47 (2.31)	0.076
Alcohol-related harm	1.67 (2.52)	1.62 (2.60)	0.40	1.71 (2.99)	1.44 (2.57)	0.052
AUDIT total	5.51 (4.56)	5.3 (4.46)	0.50	5.13 (4.55)	4.7 (4.12)	**0.014**

Bold values indicate statistical significance.

**Figure 2:**
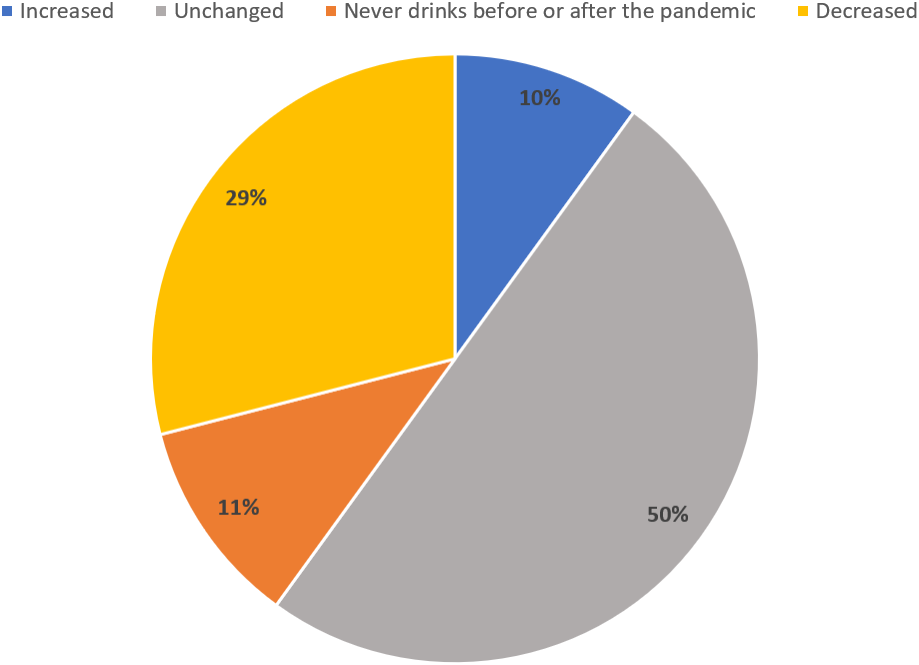
Changes in alcohol consumption since the start of the COVID-19 pandemic: The majority of respondents did not change their drinking habits (50%) since the start of the pandemic. Drinking decreased in 29% of participants while an increase was reported in 10% of the sample. 11% never drank before or after the COVID-19 pandemic N=1067.

Likewise, high alcohol consumption decreased by two percentage points from 33.1% in wave 3 to 31.1% in wave 4. Perceived changes in alcohol consumption since the start of the COVID-19 pandemic revealed a decrease in alcohol use in 29% of participants compared to 10% who reported increasing their drinking. Half of the participants (50%) did not change their alcohol consumption (**Figure 2**).

### 3.2 Alcohol use and changes in demographics since the start of the COVID-19 pandemic

Shifts in working/learning place during the COVID-19 pandemic were associated with perceived changes in alcohol consumption. Interestingly, decreased drinking was 1.4 fold more likely with remote working/studying while increased drinking had a likelihood ratio of 1.5:1 in those working/studying in their regular workplace p = 2×10^-6^ (**Table 2**) Increased drinking was 1.7 fold more common among those who experienced longer working hours compared to those with unchanged or reduced working time (p = 0.03 **Table 2**). Worsened financial situations were 1.7 fold more likely among those who increased their alcohol use compared to participants who reduced their alcohol use p = 0.02 (**Table 2**). Being laid off or unemployed were not associated with changes in drinking habits (p > 0.05).

**Table 2:** Associations between retrospective cross-sectional changes in alcohol use and changes in demographics, social, and mental health factors during the COVID-19 pandemic.

Factors		Increased consumption N, %	Unchanged N, %	Decreased consumption N, %	*P*
Demographics					
Working conditions	Remote working	24, 24.5%	98, 19.4%	101, 33.7%	**2×10^-6^**
	Regular workplace	29, 29.6%	190, 37.5%	60, 20%	
	Both	45, 45.9%	218, 43.1%	139, 46.3%	
Working hours	Increased	29, 28.7%	87, 16.2%	54, 17.3%	**0.03**
	Unchanged	59, 58.4%	393, 73.3%	223, 71.5%	
	Decreased	13, 12.9%	56, 10.4%	35, 11.2%	
financial situation	Worsened	24, 23.8%	81, 15.1%	44, 14.1%	**0.02**
	Unchanged	65, 64.4%	398, 74.3%	217, 69.6%	
	Improved	12, 11.9%	57, 10.6%	51, 16.3%	
Social factors					
Feelings of Loneliness	More often	51, 50.6%	165, 30.8%	164, 52.6%	**8.8×10^-10^**
	Unchanged	45, 44.6%	348, 64.9%	131, 42%	
	Less often	5, 5%	23, 4.3%	17, 5.4%	
Meetings with friends	More often	15, 14.9%	34, 6.5%	24, 7.7%	**4.8×10^-11^**
	Unchanged	29, 28.7%	240, 44.7%	69, 22%	
	Less often	57, 56.4%	262, 48.9%	220, 70.3%	
Mental health					
Anxiety	Increased	30, 30%	101, 20.4%	101, 33.4%	**1.2×10^-4^**
	Unchanged	61, 61%	367, 74.1%	177, 58.6%	
	Decreased	9, 9%	27, 5.5%	24, 7.9%	
Depression	Increased	42, 43.8%	117, 27.1%	122, 44%	**3.3×10^-8^**
	Unchanged	41, 42.7%	283, 65.7%	121, 43.7%	
	Decreased	13, 13.5%	31, 7.1%	34, 12.3%	

Bold values indicate statistical significance.

### 3.3 Changes in social factors and alcohol consumption

Our analysis illustrated that changes in alcohol consumption were 1.6 fold more likely in those who experienced an increase in loneliness compared to unchanged alcohol consumption p = 8.8×10^-10^. The majority of those with unchanged alcohol use also reported unaffected feelings of loneliness (65%). Decreased feelings of loneliness had the smallest percentages across all three groups (**Table 2**). Moreover, perceived changes in alcohol consumption were associated with changes in meetings with friends p = 4.8×10^-11^, (**Table 2**). Those who increased their consumption were roughly two-fold more likely to report meeting with their friends more often (14.9%) compared to those who reduced their consumption (7.7%). Likewise, unchanged alcohol consumption was mostly among those with unchanged or reduced instances of meeting with friends (44.7% and 48.8% respectively).

### 3.4 Alcohol use and changes in mental health

The majority of participants reported unchanged feelings of anxiety. Nonetheless, shifts in perceived changes in alcohol consumption, either increased or decreased, were roughly 1.6 fold more likely in those who experienced a rise in feelings of anxiety p= 1.2×10^-4^ compared to unchanged alcohol consumption. In contrast, reduced feelings of anxiety were the least reported outcome during the pandemic across all groups. Regarding feelings of depression, participants who reported a change in their alcohol consumption were 1.6 fold more likely to report an increase in feelings of depression compared to those with unchanged alcohol use 3.3×10^-8^. On the other hand, those with unchanged consumption were 1.7 fold more likely to experience a decrease in feelings of depression (**Table 2**).

Our study revealed a noteworthy resemblance in the trend of individuals who either increased or decreased their alcohol consumption in regard to feelings of anxiety/depression during the pandemic (**Table 2**). To understand the interplay between these variables, we sought to investigate the relationship between social isolation/loneliness, depressive feelings, and alcohol consumption among young adults. Unsurprisingly, participants who reported feeling lonely more often had a likelihood ratio of 3:1 to report increased feelings of depression while those who felt less lonely during the pandemic had a likelihood ratio of 3.4:1 to report meetings with their friends more often. However, when using a univariate linear model to assess the relationship with alcohol-related problems, increased loneliness was associated with higher alcohol-related harm scores while those who felt less lonely reported less alcohol-related harm. Taken together, these results suggest a differential relationship between feelings of loneliness and alcohol-related harm among young adults. Both anxiety and depressive symptoms were positively associated with increased alcohol-related harm, p < 0.001 (**Table 3**).

**Table 3:** Univariate analysis on the cross-sectional association between psychiatric traits and alcohol-related harm in the SALVe cohort at wave 4 controlled for sex.

Psychiatric trait	*β*	*P*
Depression	0.19	**5.4×10^-8^**
Anxiety	0.05	**3×10^-7^**
Loneliness	0.34	**0.004**

Bold values indicate statistical significance.

### 3.5 Pre-pandemic predictors of changes in alcohol consumption

we expanded our analysis to identify pre-pandemic predictors of harmful alcohol use following the COVID-19 pandemic (**Table 4**). After adjusting for sex, ADHD and delinquency symptoms were associated with harmful use following the pandemic (Odds ratio= 1.02 and 1.26, p = 7.4×10^-4^ and 1.6×10^-4^ respectively). These associations remained after accounting for the rest of the pre-pandemic traits, p= 0.003, and 0.02 respectively.

**Table 4:** Longitudinal association between pre-pandemic traits at wave 3 and harmful alcohol consumption following the COVID-19 pandemic in the SALVe cohort adjusted for sex.

Pre-pandemic trait	*Odds ratio*	*95% OR CI*		*P*	P*
		Lower	Upper		
Depression symptoms	1.04	0.99	1.10	0.07	0.97
Anxiety symptoms	1.01	0.99	1.03	0.13	0.79
Delinquency symptoms	1.26	1.12	1.42	**1.6×10^-4^**	**0.003**
ADHD symptoms	1.02	1.009	1.034	**7.4×10^-4^**	**0.02**
Psychotic-like symptoms	1.01	0.95	1.07	0.76	0.38
Drug use	1.05	0.99	1.1	0.06	0.35

*adjusted for the rest of the pre-pandemic traits, Harmful consumption was classified following the WHO recommendations (male ≥ 8 and female ≥ 6).

Bold values indicate statistical significance.

## 4 Discussion

This study describes changes in alcohol use, contributing factors, and predictors of hazardous consumption during the COVID-19 pandemic. Alcohol consumption showed both a decrease and increase among young adults. Roughly half of the participants did not change their alcohol use habits throughout the COVID-19 pandemic. Furthermore, reduced consumption was reported roughly three-fold more frequently than an increase in alcohol use. Alcohol consumption remained unchanged during the COVID-19 pandemic similar to a previous study during the first months in young adults (19). The present study, however, used a longer follow-up period covering the different policies implemented during the pandemic until October 2021. Sex-stratified analysis revealed that only females reduced harmful alcohol use since the start of the COVID-19 pandemic. This result is particularly important given the lack of longitudinal data and the mixed results on sex differences, especially among young adults (17). However, our results are consistent with research early in the pandemic indicating that young women in particular decreased risky consumption during the pandemic (14).

Interestingly, we report two distinct groups that were more likely to increase their consumption. First, young adults who managed to meet with their friends more often and felt less lonely were more likely to increase their consumption. The second group consists of those who felt an increase in feelings of anxiety, loneliness, and depression, leading to an increase in alcohol consumption. In addition, participants who continued to work/study from their regular workplace were more likely to increase their consumption compared to remote work/learning.

The Swedish response to the COVID-19 pandemic differed considerably from other countries and even to the other Nordic nations. The Swedish public health agency (Folkhälsomyndigheten) did not impose large lockdowns and instead, focused on voluntary individual responsibilities. Public gatherings were limited in the first wave of the pandemic and universities were advised to shift to remote learning (37). Bars and restaurants remained open throughout 2021 but had to close at earlier hours before all restrictions were finally lifted in September 2021 (38). Despite the relaxed policy, the present study found that alcohol use and alcohol-related harm have decreased among Swedish young adults since the start of the COVID-19 pandemic. Cohort studies focusing on the trend of alcohol use among young adults throughout the pandemic are limited. Nevertheless, in contrast to our analysis, a longitudinal study in Belgium, indicated that while participants, particularly young adults, decreased their consumption during the lockdown period, they returned to initial consumption levels after the lockdown offset (23). However, our study shares similar findings to a large study on the trend of alcohol consumption over six years in the U.S (up to Nov 2020, the first year of the COVID-19 pandemic) that demonstrated an increase in drinking alone following the pandemic with drinking reasons such as drinking to relieve tension and due to boredom also deviating upwards in 2020 (39). Moreover, depression and anxiety symptoms were also associated with higher alcohol use (39) consistent with our findings, and previous literature (17). Both studies, however, were only limited to only the first year of the pandemic compared to the present analysis.

**Figure 3:**
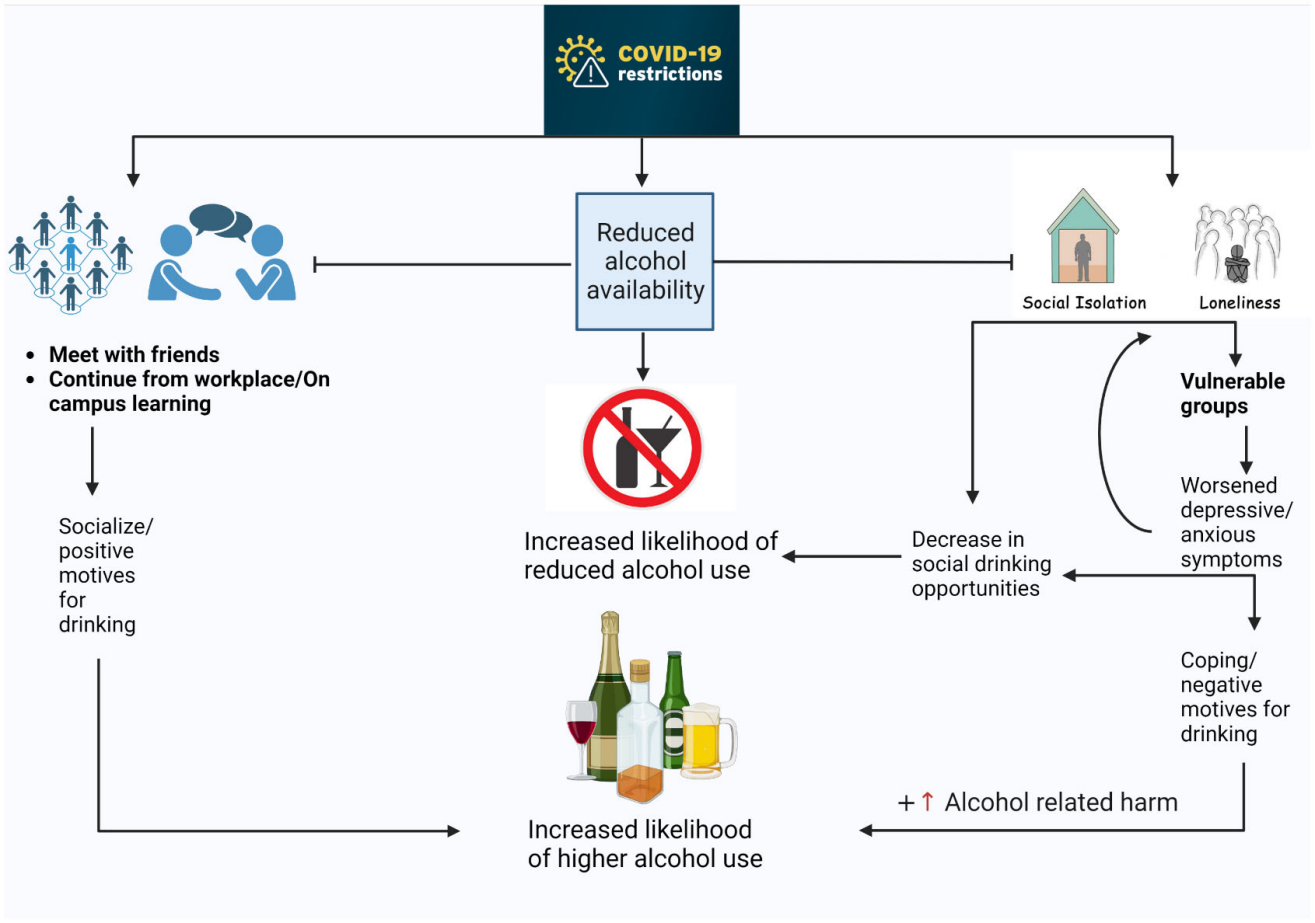
Theoretical model for the patterns of alcohol consumption among young adults during the COVID-19 pandemic: The COVID-19 pandemic disrupted the social life and emotional well-being of young adults leading to increased distress and social isolation (*Mental Health and COVID-19: Early evidence of the pandemic’s impact: Scientific brief, 2 March 2022*). The public health response has affected both the physical and the financial availability of alcohol as well as drinking opportunities (10). These effects may lead to a decrease in overall alcohol consumption. However, vulnerable groups and those with pre-existing conditions may suffer from worsening depressive/anxiety symptoms and loneliness (5). Eventually, this may result in increased alcohol use as a way to cope with the pandemic. Consequentially, the burden of alcohol-related harm may surge in such subgroups (17, 39). On the other hand, some young adults who are less affected by the pandemic, meeting with their friends more often and continuing their work/studies from their regular workplace without interruption, may be motivated to increase their drinking to socialize and achieve a positive outcome (12, 41, 43).

On the other hand, young adults who met their friends more often following the pandemic were approximately two-fold more likely to increase their alcohol consumption. Furthermore, those who continued to work/study from their regular workplace were also more likely to increase their consumption compared to remote work/learning. Interestingly, the two distinct aspects of higher alcohol consumption during the pandemic had opposite effects on feelings of loneliness. While both were associated with the likelihood of increased consumption, those with worsened feelings of depression had a likelihood ratio of 3:1 to experience an increase in feelings of loneliness. On the contrary, decreased feelings of loneliness were 3.4 fold more likely among those who socialized more often with friends. Few studies explored the strength of the relationship between loneliness and alcohol use. However, recent results have shown that loneliness and indicators of well-being do not differ significantly by frequency of alcohol consumption (40). Previous literature has highlighted the role of socialization on the drinking habits of young adults (12, 13, 41–43). Indeed, a recent study in Sweden using a nationally representative sample has shown that social motives are the most common motives for alcohol use among Swedish adolescents and were associated with a higher frequency of alcohol consumption (43). Likewise, the present study may be interpreted by the differences in individual experiences during the pandemic (**Figure 3**). The subgroup of young adults who were less affected by the pandemic, had an increase in their meetings with friends and continued to work/study from their regular workplace, had an increased likelihood of higher alcohol consumption. Notably, alcohol-related harm was positively associated with increased levels of loneliness illustrating a difference in the consequences of social drinking as opposed to drinking to cope with anxious/depressive feelings. Nonetheless, our interpretation of the results is limited by the lack of data on drinking motives during the pandemic in the SALVe cohort.

In addition, the present analysis showed that anxiety and depression symptoms were associated with higher alcohol-related harm cross-sectionally. Interestingly, both symptoms at wave 3 did not predict hazardous consumption following the start of the COVID-19 pandemic. This is particularly significant due to the increase in depression and anxiety during the COVID-19 pandemic. These results are in contrast to a follow-up study at the start of the pandemic that indicated both depression and anxiety were predictors of hazardous alcohol consumption in young adults (44). The study was done during the first phase of the pandemic and the follow-up interval was limited only three months compared to the present analysis. Predictors of harmful alcohol consumption during the pandemic were pre-pandemic ADHD and delinquency symptoms. These results are consistent with previous studies showing prior harmful alcohol use, impulsivity, and antisocial problems as risk factors for alcohol-related harm (45–47).

The only discrepancy in outcomes observed in this study is in the subgroup reporting reduced feelings of depression and loneliness alongside higher alcohol consumption. These results may be attributed to the social context surrounding youth drinking (**Table 2**). While only a limited number of studies have examined the impact of COVID-19 on alcohol use using data that covers both the period before and throughout the pandemic, such studies have reported various shifts in alcohol consumption patterns (48). The present study contributes further to the knowledge concerning youth alcohol use and risk patterns during the COVID-19 pandemic by leveraging the comprehensive data available in the SALVe cohort, which includes information on social factors, mental health, and prior alcohol and drug use.

The strength of the present study lies in the randomized longitudinal design with large sample size, the added variables on the psychosocial and work environment changes among young adults to explore factors that contributed to shifts in alcohol use throughout the COVID-19 pandemic, and the comprehensive data on mental health to evaluate predictors of harmful alcohol consumption. Nonetheless, the study has several limitations. First, the unique approach to the COVID-19 pandemic in Sweden as well as the geographically restricted nature of the SALVE sample (Västmanland) limit comparisons as factors like alcohol availability and social opportunities differed significantly. Additionally, the SALVe cohort lacked data on drinking motives during the pandemic and therefore, results should be interpreted with caution. Likewise, part of the analyses relied on retrospective reports that are subject to recall bias and memory inaccuracies. Moreover, while the two time points for data collection enabled a better understanding of the trend of alcohol use following the pandemic, the use of multiple time points reflecting the easing and restricting of COVID-19 measurements may have contributed to a better comprehension of the acute shifts of alcohol use. Finally, as the present study is in a young population, changes in drinking patterns may also be attributed to the natural development related to the transition from emerging adulthood to young adulthood, independent of the pandemic.

## 5 Conclusion

The present study found that alcohol consumption and alcohol-related harm have decreased since the start of the COVID-19 pandemic. The study found two distinct groups associated with increased alcohol consumption during the pandemic. Worsened feelings of loneliness, depression and anxiety, were more likely among those who increased their alcohol use. Similarly, those who had the opportunity to meet their friends more often, felt less lonely but had an increased likelihood of higher alcohol consumption. However, alcohol-related harm was positively associated with increased levels of loneliness. These findings provide important and timely insight into the complexity of the relationship between loneliness and alcohol use. Predictors of harmful consumption were pre-pandemic hazardous use, ADHD symptoms, and delinquency symptoms.

Our findings have some research and clinical implications. Future studies investigating the relationship between loneliness and alcohol consumption should account for the associated consequences of decreased loneliness. For example, factors such as drinking to socialize and to find a partner may similarly contribute to higher alcohol consumption as worsened feelings of loneliness. Young groups may also benefit from advice about the pros and cons of alcohol use in relations to socializing, and psychosocial well-being.

## Data availability statement

The raw data supporting the conclusions of this article will be made available by the authors, without undue reservation.

## Ethics statement

The studies involving humans were approved by Ethical Review Board in Uppsala, Dnr: 2012/187. The studies were conducted in accordance with the local legislation and institutional requirements. The participants provided their written informed consent to participate in this study.

## Author contributions

MM and GK contributed to the conceptualization, design, and data analysis of the study. KN and HS contributed to the conceptualization, design, and supervision. KN, CÅ, SV and SO developed the design for the whole SALVe cohort project. KN, CÅ, and SV administrated the data collection. KN, CÅ, and SV provided funding acquisition and project management. MM wrote the original draft and all authors contributed to the writing and critically revised the text. All authors contributed to the article and approved the submitted version.

## Footnotes

1 Spence, 2017. The Adult Anxiety Scale-15 (AAS-15), self-report version. Personal communication.
